# *Weissella cibaria* WIKIM28 ameliorates atopic dermatitis-like skin lesions by inducing tolerogenic dendritic cells and regulatory T cells in BALB/c mice

**DOI:** 10.1038/srep40040

**Published:** 2017-01-09

**Authors:** Seul Ki Lim, Min-Sung Kwon, Jieun Lee, Young Joon Oh, Ja-Young Jang, Jong-Hee Lee, Hae Woong Park, Young-Do Nam, Myung-Ji Seo, Seong Woon Roh, Hak-Jong Choi

**Affiliations:** 1 Microbiology and Functionality Research Group, World Institute of Kimchi, Gwangju 61755, Republic of Korea; 2 Advanced Process Technology and Fermentation Research Group, World Institute of Kimchi, Gwangju 61755, Republic of Korea; 3 Research Group of Gut Microbiome, Korea Food Research Institute, Seongnam 13539, Republic of Korea; 4 Division of Bioengineering, Incheon National University, Incheon 22012, Republic of Korea; 5 Biological Disaster Analysis Group, Korea Basic Science Institute, Daejeon 34133, Republic of Korea

## Abstract

The occurrence of atopic dermatitis (AD), a chronic inflammatory skin disease, has been increasing steadily in children and adults in recent decades. In this study, we evaluated the ability of the lactic acid bacterium *Weissella cibaria* WIKIM28 isolated from gatkimchi, a Korean fermented vegetable preparation made from mustard leaves, to suppress the development of AD induced by 2,4-dinitrochlorobenzene in a murine model. Oral administration of *W. cibaria* WIKIM28 reduced AD-like skin lesions, epidermal thickening, and serum immunoglobulin E levels. Furthermore, the production of type 2 helper T (Th2) cytokines such as interleukin (IL)-4, IL-5, and IL-13 decreased in peripheral lymph node cells. Moreover, the intake of *W. cibaria* WIKIM28 increased the proportion of CD4^+^CD25^+^Foxp3^+^ regulatory T (Treg) cells in mesenteric lymph nodes (MLNs) and IL-10 levels in polyclonally stimulated MLN cells. In conclusion, the oral administration of *W. cibaria* WIKIM28 isolated from gatkimchi ameliorated AD-like symptoms by suppressing allergic Th2 responses and inducing Treg responses. These results suggest that *W. cibaria* WIKIM28 may be applicable as a probiotic for the prevention and amelioration of AD.

Kimchi is a traditional Korean fermented food made using various vegetables such as Chinese cabbage, mustard leaves, or radish as the main ingredient. Kimchi undergoes lactic acid fermentation during storage, in which diverse lactic acid bacteria (LAB) are involved. *Weissella* spp. are present in different fermented vegetable-based foods and sausages, as well as in the normal gastrointestinal tract of mammals, including humans. On the basis of isolation from human faces, acid tolerance, and attachment to intestinal epithelial cells^1^,^2^, *Weissella cibaria* strains have been proposed as potential probiotics. The administration of probiotic bacteria is being considered a promising strategy to improve host health via the immune system. Numerous reports have documented the role of strain-specific LAB in the prevention of allergic disorders such as atopic dermatitis (AD), obesity, and malnutrition^3^,^4^,^5^. Although probiotics have beneficial effects towards host health, the immunomodulatory mechanisms underlying these effects remain to be elucidated.

In the last few decades, the occurrence of AD, a chronic inflammatory skin disease, has been steadily increasing in children and adults in industrialized countries. AD results from an overreacting immune system in response to foods, chemicals, and environmental factors, and it is accompanied by various symptoms such as itchy, red, swollen, and cracked skin^6^. AD is characterized by an imbalance of type 1 helper T (Th1) and type 2 helper T (Th2) cell responses, with a shift toward a predominantly Th2 response. Commonly, Th2 responses mediate immunoglobulin E (IgE) production through the release of cytokines such as interleukin (IL)-4, IL-5, and IL-13 in the AD-like murine model^7^. Recently, regulatory T cells (Tregs) in AD have been estimated to play an essential role in controlling immune tolerance by balancing the immune response in inflammation and are characterized by the dominant induction of forkhead box protein 3 (Foxp3)^7^. In the AD-like murine model, Tregs secrete IL-10^8^, which regulates the Th2 response to allergens and maintains functional tolerance^9^.

While most AD therapies entail the use of anti-inflammatory drug such as topical corticosteroids, currently, there is no cure for AD. Moreover, these drugs, which temporarily alleviate AD symptoms, can produce side effects such as skin atrophy, steroid-induced rosacea or perioral dermatitis, etc.^10^ Therefore, it is important to find novel therapeutic agents for the treatment of AD. In the present study, we isolated a *W. cibaria* strain, WIKIM28, from gatkimchi (a subtype of kimchi made from mustard leaves). This strain showed the ability to inhibit allergic reaction by suppressing Th2 responses and inducing Treg responses. We investigated the ameliorative effects of *W. cibaria* WIKIM28 on AD-like symptoms by enhancing the frequency and function of Tregs in a murine AD model.

## Results

### Ameliorative effects of the oral administration of *W. cibaria* WIKIM28 on DNCB-induced AD mice

**Figure 1:**
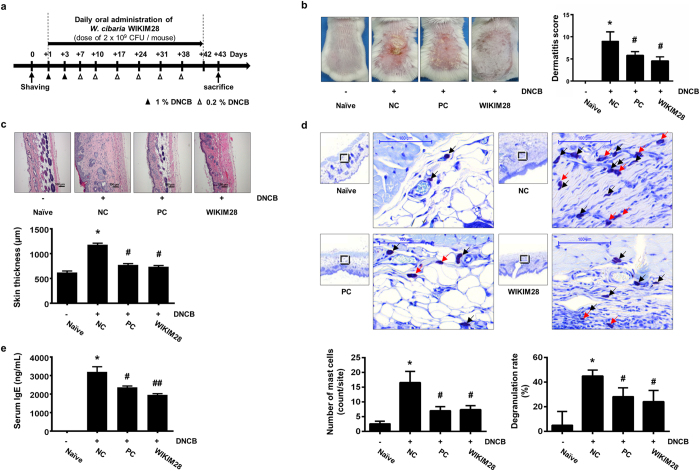
Oral administration of *W. cibaria* WIKIM28 ameliorates AD-like symptoms. (**a**) Schematic diagram of the study protocol. Mice were divided into 5 groups (n = 5 per group). To induce AD-like immunologic and skin lesions, DNCB was applied to the dorsal skin as described in the materials and methods. *W. cibaria* WIKIM28, kerotifen, or PBS were administered by intragastric gavage of a 200 μL volume once daily. (**b**) AD-like skin lesions were evaluated by visual observation. Dermatitis scores were defined as the sum of scores for five symptoms: erythema/hemorrhage, edema/excoriation, erosion, scarring/dryness, and lichenification. (**c**) Paraffin-embedded sections of skin samples from AD mice were stained with H&E and the skin thickness was measured, Bar = 100 μm. (**d**) Paraffin-embedded sections of skin samples from AD mice were stained with toluidine blue and the number of mast cells (indicated by the arrow in black) was counted, Bar = 100 μm. The rate of degranulating or degranulated mast cells (indicated by the arrow in red) among total mast cells was analyzed. (**e**) Serum IgE levels were detected by sandwich ELISA. Bars are from 5 mice per group and representative of three independent experiments. Error bars indicate SE. *p < 0.001, compared to the naïve group; ^#^*p* < 0.05, ^##^*p* < 0.001, compared to the NC group.

To investigate whether WIKIM28 has the potential to ameliorate AD symptoms in AD-like mice, we evaluated the effects of oral administration of WIKIM28 in an AD-like murine model. AD-like lesions were induced by repeated topical application of 2,4-dinitrochlorobenzene (DNCB) to the dorsal skin of BALB/c mice, and the effects of WIKIM28 were studied in groups orally administered PBS (naïve and NC group), kerotifen (anti-histamine agent; PC group), or *W. cibaria* WIKIM28 (WIKIM28 group). The schematic experimental procedure is described in Fig. 1a. We found that the oral administration of *W. cibaria* WIKIM28 significantly ameliorated the development of AD-like skin lesions compared to the NC group. Dermatitis scores of AD-like skin lesions were significantly reduced in the WIKIM28 group and PC group compared with the NC group which showed four dermatitis symptoms of erythema/hemorrhage, edema/excoriation, erosion, and scarring/dryness (Fig. 1b). Similar effects were also observed on hematoxylin and eosin (H&E) staining of sections and local infiltration of mast cells in AD-induced dorsal skin lesions. According to Kim, *et al*.^11^, edema and dense infiltration of mast cells occur in the skin layers of AD mouse models. In the present study, the WIKIM28 group exhibited suppressed edema, i.e., thickening of the epidermis caused by repeated immune response of AD-like skin lesions (Fig. 1c). The local infiltration and degranulation of mast cells also increased in the NC group. However, oral administration of kerotifen or WIKIM28 significantly suppressed the infiltration and degranulation rate of mast cells in AD-like skin lesions (Fig. 1d). We also determined the ameliorative effect of WIKIM28 on serum IgE levels, because the overproduction of serum IgE is a typical characteristic of AD symptoms and is due to the strong polarization of the Th2 immune response. As shown in Fig. 1e, the serum IgE level was significantly decreased in WIKIM28 groups compared with the NC group. In particular, the WIKIM28 group exhibited more effectiveness against AD-like symptoms than the PC group.

### Effect of cytokine production in PLN cells by oral administration of *W. cibaria* WIKIM28 on DNCB-induced AD mice

**Figure 2:**
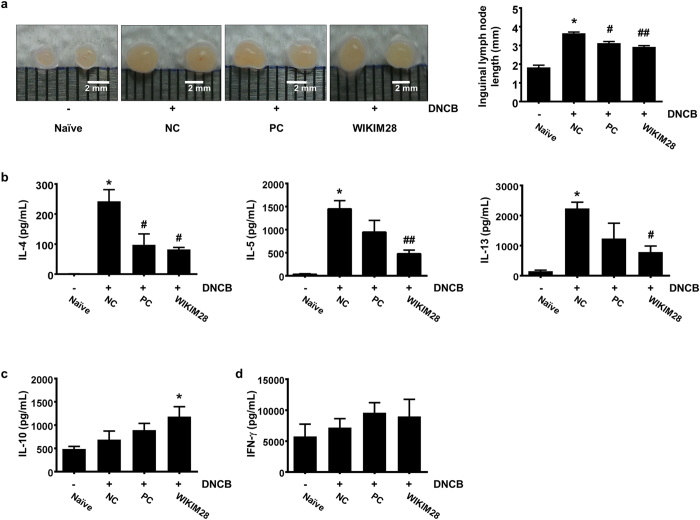
Oral administration of *W. cibaria* WIKIM28 decreases the secretion of IL-4, IL-5, and IL-13 and increased the production of IL-10 by PLN cells of AD-induced mice. (**a**) Inguinal lymph node lengths were measured. After PLN cells were treated with anti-CD3/CD28 mAbs for 24 h, the production of IL-4, IL-5, IL-13 (**b**), IL-10 (**c**), and IFN-γ (**d**) in the supernatant was detected by the Cytometric Bead Array kit. Bars are from 5 mice per group and representative of three independent experiments. Error bars indicate SE. *p < 0.001, compared to the naïve group; ^#^*p* < 0.05, ^##^*p* < 0.001, compared to the NC group.

Lymphoid organs, such as lymph nodes, near the AD-like skin lesions are generally in charge of antigen presentation, lymphocyte differentiation, and proliferation for prompt and effective elimination of antigens. The inguinal and axillary lymph nodes are located the most near the AD-like skin lesions. For these reasons, lymph nodes are enlarged in the case of dorsal dermatitis. Thus, we isolated axillary lymph nodes and inguinal lymph nodes near the inflammatory sites to measure the enlargement of organs. Oral administration of kerotifen or WIKIM28 significantly suppressed inguinal lymph node enlargement (Fig. 2a), but no change in axillary lymph node size. To address whether oral administration of WIKIM28 regulates the suppression of Th2 response at the local inflammatory sites, peripheral lymph node (PLN) cells were re-stimulated with anti-CD3/CD28 mAbs for 1day, and the levels of cytokines in the supernatant were measured. As shown in Fig. 2b,c, the secretion of IL-4, IL-5, and IL-13 cytokines was significantly decreased, and in particular, the production of IL-10 was significantly enhanced only in the WIKIM28 group compared with all other groups. Whereas, the release of interferon (IFN) -γ was slightly increased, but not significant (Fig. 2d). These results indicate that WIKIM28 induces the suppression of Th2 response and the production of IL-10, which suppresses the immune responses.

### Frequency of Tregs by oral administration of *W. cibaria* WIKIM28 in DNCB-induced AD mice

**Figure 3:**
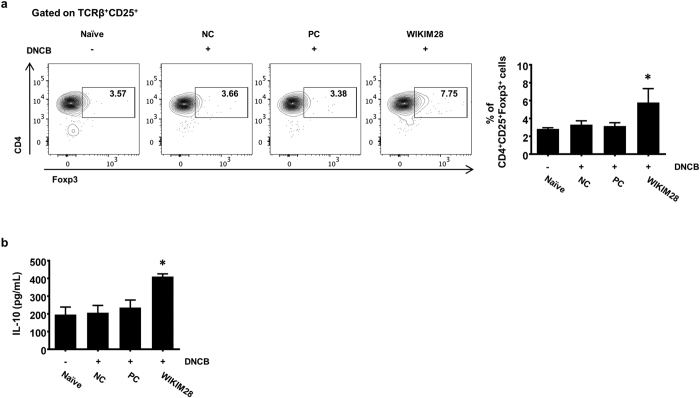
Oral administration of *W. cibaria* WIKIM28 increases the frequency of Tregs in MLNs and the production of IL-10 by MLN cells in AD-induced mice. (**a**) The CD4^+^CD25^+^Foxp3^+^ Treg proportion in MLNs was analyzed by flow cytometry. After MLN cells were treated with anti-CD3/CD28 mAbs for 24 h, (**b**) IL-10 levels in the supernatant were detected by the Cytometric Bead Array kit. Bars are from 5 mice per group and representative of three independent experiments. Error bars indicate SE. *p < 0.05, compared to the naïve group.

Probiotics are live microorganisms that, when administered in sufficient amounts, confer health benefits on the host. Ingested probiotics has been considered to affect the activity of the gut mucosal immune system. In fact, antigens (probiotics and probiotic-derived products) taken up by M cells are delivered to antigen presenting cells (APCs), mainly macrophages and dendritic cells (DCs). Antigen-loaded DCs travel to mesenteric lymph nodes and present antigens to T and B cells. Secretion of cytokine and state of activation of APCs determine whether naïve T lymphocytes differentiate into Th1, Th2 or Treg cells. Therefore, we attempted to determine whether WIKIM28 could specifically upregulate the frequency and function of Tregs in in the mesenteric lymph nodes (MLNs) of AD-like mice. The intake of WIKIM28 showed a significant increase in the proportion of CD4^+^CD25^+^Foxp3^+^ Tregs in MLNs (Fig. 3a), and the IL-10 production in polyclonally stimulated MLN cells was also significantly enhanced compared with all other groups (Fig. 3b). These results indicate that WIKIM28 induces the differentiation of Tregs and the production of IL-10, which suppresses the immune responses.

### Immunomodulatory effects of *W. cibaria* WIKIM28 on murine DCs

**Figure 4:**
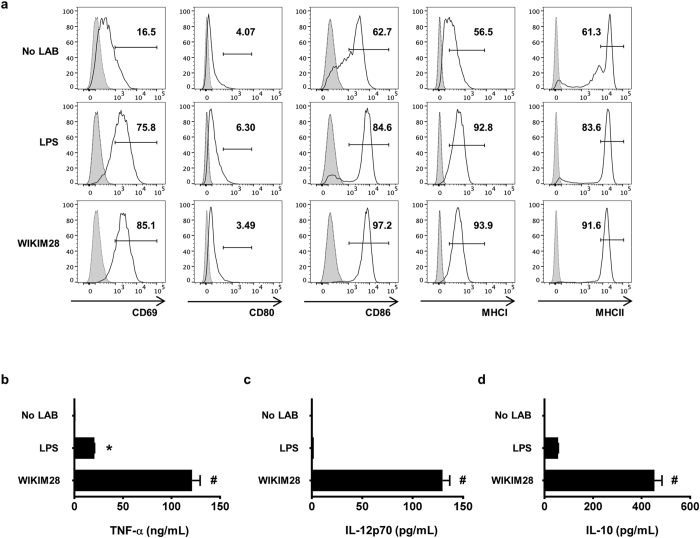
*W. cibaria* WIKIM28 isolated from gatkimchi activate BMDC functions. (**a**) The cellular activation by BMDCs in response to live gatkimchi LAB or LPS (100 ng/mL) was analyzed by flow cytometry. The release of TNF-α (**b**), IL-12p70 (**c**), and IL-10 (**d**) by BMDCs in response to WIKIM28 or LPS for 24 h was measured. Cytokines in the supernatant were detected by a Cytometric Bead Array kit. Error bars indicate SE. Data are representative of three independent experiments. *p < 0.05, ^#^*p* < 0.001, compared to the negative control group (No LAB).

**Figure 5:**
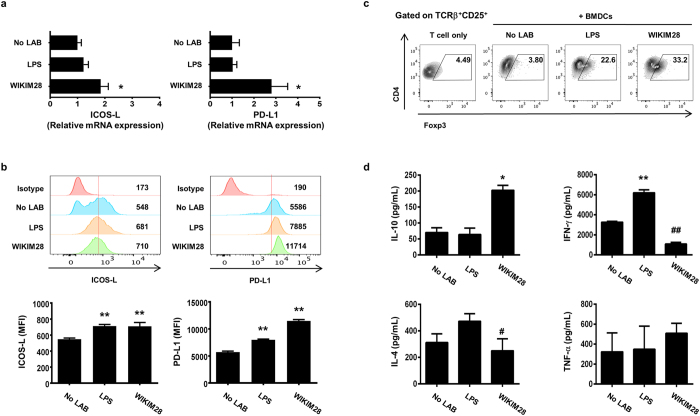
*W. cibaria* WIKIM28 isolated from gatkimchi enhance the gene expression of tolerogenic DC markers and the proportion and function of Tregs. (**a,b**) The expression levels of ICOS-L and PD-L1 in BMDCs were measured by real-time PCR (**a**) and flow cytometry (**b**). The mean fluorescence intensity (MFI) of PD-L1 and ICOS-L on BMDCs. (**c,d**) After incubation of BMDCs with WIKIM28, activated DCs were co-cultured with naïve CD4^+^ T cells for 5 days on anti-CD3/CD28 mAbs-coated plates. The CD4^+^CD25^+^Foxp3^+^ Treg proportion was then analyzed by flow cytometry (**c**). IL-10, IFN-γ, IL-4, and TNF-α production in the supernatant was quantitated by flow cytometry using a Cytometric Bead Array kit (**d**). Error bars indicate SE. Data are representative of three independent experiments. *p < 0.05, **p < 0.001, compared to the negative control group (No LAB). ^#^*p* < 0.05, ^##^*p* < 0.001, compared to the LPS.

Although LAB are well documented to possess immunomodulatory effects on host health^12^, the mechanisms underlying these effects remain to be clarified. As antigen-presenting cells, DCs allow for enhanced activation of T cells and determination of the Th1/Th2 polarization upon stimulation^13^,^14^. To evaluate whether the function of DCs is affected by WIKIM28, we attempted to treat murine bone marrow-derived dendritic cells (BMDCs) with WIKIM28. As shown in Fig. 4a, WIKIM28-treated BMDCs expressed high levels of the activation markers CD69, CD80, CD86, and MHCII as compared to unstimulated BMDCs. In addition to BMDC activation, the cytokine secretion by mature DCs, including tumor necrosis factor (TNF) -α, IL-12p70, and IL-10, was measured. As shown in Fig. 4b–d, WIKIM28 increased the TNF-α secretion compared with lipopolysaccharide (LPS) -stimulated or unstimulated BMDCs, and the release of IL-12p70 and IL-10 was markedly increased by WIKIM28 treatment. We next investigated the role of DCs by measuring the gene expression of surface molecules in LAB-treated BMDCs. WIKIM28 significantly upregulated the mRNA and surface expression levels of ICOS-L and PD-L1, which are tolerogenic DC markers (Fig. 5a,b). These results indicate that WIKIM28 induces the generation of activated and tolerogenic DCs, simultaneously. Therefore, we further investigated whether WIKIM28 have the potential to enhance the differentiation and function of Tregs. DCs treated with WIKIM28 were co-cultured with naïve CD4^+^ T cells for 5 days on anti-CD3/CD28 mAbs-coated plates, and the CD4^+^CD25^+^Foxp3^+^ Treg proportion and cytokines secretion was analyzed by flow cytometry using a Cytometric Bead Array kit. The results showed that WIKIM28 strongly augmented the differentiation of CD4^+^CD25^+^Foxp3^+^ Tregs, and the secretion of IL-10 was significantly increased (Fig. 5c,d). Whereas, the production of IFN-γ and IL-4 was significantly decreased in the WIKIM28 group compared with control group, and the level of TNF-α secretion was comparable in all group (Fig. 5d). Taken together, these results indicate that although the phenotype of DCs treated with WIKIM28 is both activated and tolerogenic, WIKIM28-treated DCs may be functionally tolerogenic, as they promote the generation of CD4^+^CD25^+^Foxp3^+^ T cells without inducing Th1 immune responses.

## Discussion

Probiotics are nonpathogenic microorganisms that are believed to provide various health benefits when consumed^15^. Although many investigations have established the involvement of probiotics in controlling various aspects of inflammation, little information is available on the mechanism whereby administered probiotics generate Treg populations^16^.

In the present study, we isolated *W. cibaria* WIKIM28 with potent immunomodulatory effects from gatkimchi and demonstrated the mechanism by which it improves AD-like symptoms in BALB/c mice. Our results showed that clinical symptoms of DNCB-induced AD-like lesions, such as erythema/hemorrhage, edema/excoriation, erosion, scarring/dryness, and lichenification, were ameliorated, and the local accumulation and degranulation rate of mast cells were suppressed by the oral administration of WIKIM28 (Fig. 1b, d). Accordingly, the serum IgE level and Th2 response were reduced (Figs 1e and 2b). Whereas, release of IFN-γ was slightly increased, but not significant in the WIKIM28 group. A similar study reported that oral administration of probiotics inhibited the production of IL-4 and IL-5, although it decreased the secretion of IFN-γ which is capable of blocking Th2 differentiation^17^. As a suppressor of Th2 response, IL-10 is well known to suppress T cell polarization into Th2 cells producing IL-4, IL-5, and IL-13^18^. We found that WIKIM28 enhanced the production of IL-10 in PLN (Fig. 2c) and spleen (Supplementary Fig. S1). These results suggest that WIKIM28 promotes rather Treg function than the control of the Th1/Th2 balance.

To investigate the mechanism of Treg generation by WIKIM28, we analyzed the effects of WIKIM28 on DC function. DCs are a crucial immune cell subset linking the innate immune response and acquisition of immunity by their capacity to recognize pathogenic and endogenous inflammatory signals and are known to play a pivotal role in the mechanism of probiotics action in allergic diseases by inducing regulatory T cells^19^. WIKIM28-treated DCs showed increased the expression of DC activation markers (CD69, CD80, CD86, and MHCI) and level of TNF-α and IL-12p70. Interestingly, WIKIM28 also enhanced the secretion of IL-10, which is a cytokine produced by tolerogenic DCs^20^. To elucidate whether it is a normal feature that the majority of LAB are capable of increasing the release of IL-10 in DCs, IL-10 production by WIKIM28 was compared with other LAB isolated from gatkimchi. As a result, WIKIM28-treated DCs showed the highest IL-10 level compared to other LAB (Supplementary Fig. S2). These results prompted us to question whether WIKIM28 could induce the generation of tolerogenic DCs which promote Treg differentiation. As shown in Fig. 5a,b, gene and surface expression of tolerogenic markers was significantly enhanced in WIKIM28-treated DCs, indicating that WIKIM28 induces the generation of activated and tolerogenic DCs, simultaneously. According to Wu, *et al*.^2^ and Gao, *et al*.^21^, DCs with higher ICOS-L and PD-L1 expression are more efficient in inducing Tregs. Interestingly, when we co-cultured WIKIM28-treated DCs with naïve CD4^+^ T cells, the frequency of Treg was markedly increased by WIKIM28. These results suggest that although WIKIM28-treated DCs showed the phenotype of both activated and tolerogenic DCs, their ability to determine T cell function may be more similar to tolerogenic DCs. The reduced secretion of Th1 cytokines by T cells co-cultured with WIKIM28-treated DCs further supports this notion (Fig. 5d). In addition to the DCs, macrophages are abundant producer of IL-10 and play a crucial role in the regulation of intestinal immune response^22^. Therefore, it is possible that macrophage may also be involved in the generation of Tregs by oral administration of WIKIM28.

In conclusion, the oral administration of *W. cibaria* strain WIKIM28 isolated from gatkimchi ameliorated AD-like symptoms by the suppression of allergic Th2 responses and the induction of Treg differentiation and function. These results suggest a potential application of *W. cibaria* WIKIM28 as a dietary supplement or a therapeutic agent to ameliorate AD.

## Materials and Methods

### Isolation and preparation of *Weissella cibaria* WIKIM28

*Weissella cibaria* WIKIM28 was isolated from gatkimchi manufactured in the province of Jeollanam-do, Republic of Korea. First, the gatkimchi was homogenized in stomacher, and the homogenate was filtered through stomacher filter bag. This filtered homogenate was diluted by dilution and spread onto de Man, Rogosa, and Sharpe (MRS; BD Difco, Sparks, MD) agar. The plate were then incubated at 30 °C for 2 days. The resulting colony of LAB was isolated by sequential culture and identified on the basis of its 16S rRNA gene sequence. Sequence data were aligned and compared with sequences deposited in the GenBank database. The phylogenetic analysis of the 16S rRNA gene sequence showed a 99.86% similarity to that of *W. cibaria*. Therefore, we identified WIKIM28 as a strain of *W. cibaria* and deposited it at Korean Federation of Culture Collection as KFCC 11625P. *W. cibaria* WIKIM28 was cultured at 30 °C MRS broth and grown overnight. The cultures were then diluted 1:200 in fresh medium and cultured for a second night for optimal growth. The OD_600_ was measured, and the number of colony-forming units (CFU) was calculated based on standard growth curves. For all cultured bacterial strains, an OD_600_ value of 1 corresponds to 1 × 10^8^ CFU/mL, which was confirmed by plating serial dilutions on MRS agar plates. After overnight culture, bacteria were washed in fresh, sterile phosphate-buffered saline (PBS; pH 7.4) and immediately administered to the mice. The mice received either sterile PBS or 2 × 10^9^ CFU bacteria in 200 μL PBS by intragastric gavage every day.

### Animal studies

Wild-type male BALB/c mice were purchased from OrientBio Co. (Seongnam, Republic of Korea). The animals were housed at 2~3 mice per individually ventilated cage at a temperature of 22 °C ± 2 °C and a relative humidity of 55 ± 5% under a 12-h light-dark cycle in a pathogen-free animal facility at the World Institute of Kimchi. The mice were fed standard chow and had *ad libitum* access to water. To study the ameliorative effects of LAB administration on AD, AD-like lesions were induced by 2,4-dinitrochlorobenzene (Sigma-Aldrich, Saint Louis, MO) according to Kim, *et al*.^23^. Mice were randomized into 4 groups (n = 5 per group): a non-induction group (naïve group, mice fed the vehicle), negative control group (NC group, mice sensitized with DNCB and fed the vehicle), positive control group (PC group, mice sensitized with DNCB and fed the anti-histamine agent kerotifen at 1 mg/kg), and WIKIM28 group (mice sensitized with DNCB and fed *W. cibaria* WIKIM28). Dorsal skin was shaved, and 200 μL of 1% DNCB in acetone/olive oil (3:1) was applied to the dorsal skin twice a week. Three weeks after the first induction, 0.2% DNCB was applied to the dorsal skin once a week. WIKIM28 or PBS (200 μL) were administered by intragastric gavage once daily. The vehicle and LAB were administered for 42 days. On day 43, the mice were sacrificed, after which the blood, dorsal skin, spleens, MLNs, and PLNs were removed for further analysis. All animal procedures were performed following the National Institutes of Health Guidelines for the Humane Treatment of Animals, with approval from the Institutional Animal Care and Use Committee of the World Institutes of Kimchi (WIKIM IACUC 201509). All sacrifices were performed under CO_2_ condition, and all efforts were made to minimize suffering.

### Evaluation of dermatitis

The severity of the dermatitis in the dorsal skin of DNCB-induced AD mice was evaluated. The severity of five symptoms (erythema/hemorrhage, edema/excoriation, erosion, scarring/dryness, and lichenification) was scored as 0 (none), 1 (mild), 2 (moderate), or 3 (severe). Dermatitis score was defined as the sum of these individual scores in accordance with the method of Matsuda *et al*.^24^.

### *In vitro* culture and stimulation of murine BMDCs

Bone marrow (BM) cells were isolated and cultured as described by Lutz, *et al*.^25^. Femora and tibiae from 6-week-old male BALB/c mice were removed and stripped of muscles and tendons. The bones were rinsed in PBS and then carefully crushed with a mortar to release the BM cells. After washes with RPMI-1640 medium, BM cells (2 × 10^6^) were seeded into petri dishes in 10 mL complete RPMI-1640 (supplemented with 10% (v/v) fetal bovine serum, 100 U/mL penicillin, 100 μg/mL streptomycin, and 50 μM β-mercaptoethanol) in the presence of 20 ng/mL murine granulocyte-macrophage colony-stimulating factor (GM-CSF; Peprotech, Rocky Hill, NJ). The cells were incubated for 8 days at 37 °C. On day 3, culture medium was supplemented with fresh complete RPMI-1640 containing 20 ng/mL murine GM-CSF. On day 8, culture medium was replaced with fresh complete RPMI-1640 containing 20 ng/mL murine GM-CSF. On day 10, immature dendritic cells (DCs) were collected and seeded in a 96-well plate at 5 × 10^5^ cells/well. Then, the cells were either left unstimulated or stimulated with *W. cibaria* WIKIM28 (1:10 cell to bacteria ratio) or LPS (100 ng/mL) for 24 h at 37 °C. After incubation, the culture supernatants were collected, and TNF-α, IL-12p70, IL-10, IL-4, and IFN-γ levels were determined by flow cytometry using a Cytometric Bead Array kit (BD Bioscience, San Jose, CA). For phenotypic analysis, cells were stained for the dendritic cell marker CD11c; the activation markers CD69, CD80, CD86, and MHCII; or appropriate isotype controls (BD Bioscience) and further analyzed by flow cytometer.

### RNA isolation, cDNA synthesis, and quantitative real-time PCR

Total RNA was isolated from the LAB-treated BMDCs using TRIzol reagent (Life Technologies, Grand Island, NY). Reverse transcription was performed with reverse transcriptase (Intron, Seongnam, Republic of Korea) primed with oligo (dT) primer. The synthesized cDNAs were amplified by quantitative real-time PCR (SYBR Green; Enzynomics, Daejeon, Republic of Korea) with an Alpha Unit Block assembly engine system (Bio-Rad, Hercules, CA) using the following primers: murine inducible co-stimulator ligand (ICOS-L) (forward) 5′-GAC TGA AGT CGG TGC AAT GGT-3′ and (reverse) 5′-TGG GTT TTC GAT TTG CCA ATA GA-3′, programmed death-ligand 1 (PD-L1) (forward) 5′-ATG CTG CCC TTC AGA TCA CAG-3′ and (reverse) 5′-TGG TTG ATT TTG CGGTAT GGG-3′, and GAPDH (forward) 5′-TGC ACC AAC TGCTTA GC-3′ and (reverse) 5′-GGA TGC AGG GAT GTT CT-3′. All PCR experiments were performed under the same cycling conditions: 95 °C for 5 min, followed by 40 cycles of 95 °C for 30 s, 58 °C for 30 s, and 72 °C for 30 s.

### Measurement of total serum IgE and cytokines

Blood samples were collected from the mice after sacrifice, and serum samples were obtained by centrifugation (3000× *g*, 10 min). Serum IgE levels were measured using ELISA OptEIA Mouse Sets (BD Bioscience). Single-cell suspensions from spleens, PLNs, and MLNs were obtained by mechanical disruption in 0.5 mL of complete RPMI-1640. The cells were seeded in a 96-well plate at 5 × 10^5^ cells/well. The cells of the spleens, PLNs, and MLNs were co-cultured with anti-CD3/CD28 monoclonal antibodies (mAbs) (1 μg) for 24 h at 37 °C. IL-4, IL-5, IL-10, IL-13, and IFN-γ production in the supernatant was quantitated by flow cytometry using a Cytometric Bead Array kit (BD Bioscience).

### Histological analysis

The dorsal skins of the experimental mice were removed, fixed in 10% phosphate-buffered formalin, and embedded in paraffin. The paraffin-embedded tissue sections were stained with hematoxylin and eosin for the evaluation of edema. The other sections were stained with toluidine blue for the detection of mast cells.

### Flow cytometric analysis

MLNs isolated from the respective groups were stained with fluorescein isothiocyanate (FITC)-labeled anti-mouse CD4 mAb, allophycocyanin-labeled anti-mouse CD25 mAb, PerCP-Cy5.5-labeled anti-mouse TCRβ mAb (BD Bioscience), and intracellular marker phycoerythrin (PE)-labeled anti-mouse Foxp3 mAb (eBioscience, San Diego, CA). Treg generated from CD4^+^ T cells were co-cultured with LPS or *W. cibaria* WIKIM28-treated BMCDs and stained with allophycocyanin-labeled anti-mouse ICOS-L mAb (Biolegend Inc., Dedham, MA), PE-labeled anti-mouse PD-L1 mAb (BD Bioscience). Flow cytometry was performed using a FACSCanto II system (BD Bioscience), and data analysis was performed using FlowJo software (Tree Star Inc., Ashland, OR).

### Nucleotide sequence accession number

The 16S rRNA gene sequence of *W. cibaria* WIKIM28 has been deposited in GenBank with accession number KU555931.

### Statistical analysis

Statistical analysis was performed using GraphPad Prism 6.0 (GraphPad Software, La Jolla, CA), and results were presented as the mean ± the standard error (SE). Treatment effects were analyzed using one-way ANOVA. A *p* value of <0.05 was considered statistically significant.

## Additional Information

**How to cite this article**: Lim, S. K. *et al. Weissella cibaria* WIKIM28 ameliorates atopic dermatitis-like skin lesions by inducing tolerogenic dendritic cells and regulatory T cells in BALB/c mice. *Sci. Rep.* **7**, 40040; doi: 10.1038/srep40040 (2017).

**Publisher's note:** Springer Nature remains neutral with regard to jurisdictional claims in published maps and institutional affiliations.

## Supplementary Material

Supplementary Information
