# Supplementary material for: Weissella cibaria WIKIM28 ameliorates atopic dermatitis-like skin lesions by inducing tolerogenic dendritic cells and regulatory T cells in BALB/c mice
Source: Sci Rep. 2017 Jan 9;7:40040. doi: 10.1038/srep40040 (PMC5220369; doi:10.1038/srep40040)
Supplement: Supplementary Information [file srep40040-s1.pdf]

Weissella cibaria WIKIM28 ameliorates atopic dermatitis-like skin lesions by inducing tolerogenic dendritic cells and regulatory T cells in BALB/c mice

Seul Ki Lim[1†], Min-Sung Kwon[1†], Jieun Lee[1], Young Joon Oh[1], Ja-Young Jang[1], Jong-Hee Lee[1], Hae Woong Park[2], Young-Do Nam[3], Myung-Ji Seo[4], Seong Woon Roh[5], Hak-Jong Choi[1*]

[1]Microbiology and Functionality Research Group, World Institute of Kimchi, Gwangju 61755, Republic of Korea

[2]Advanced Process Technology and Fermentation Research Group, World Institute of Kimchi, Gwangju 61755, Republic of Korea

[3]Research Group of Gut Microbiome, Korea Food Research Institute, Seongnam 13539, Republic of Korea

[4]Division of Bioengineering, Incheon National University, Incheon 22012, Republic of Korea

[5]Biological Disaster Analysis Group, Korea Basic Science Institute, Daejeon 34133, Republic of Korea

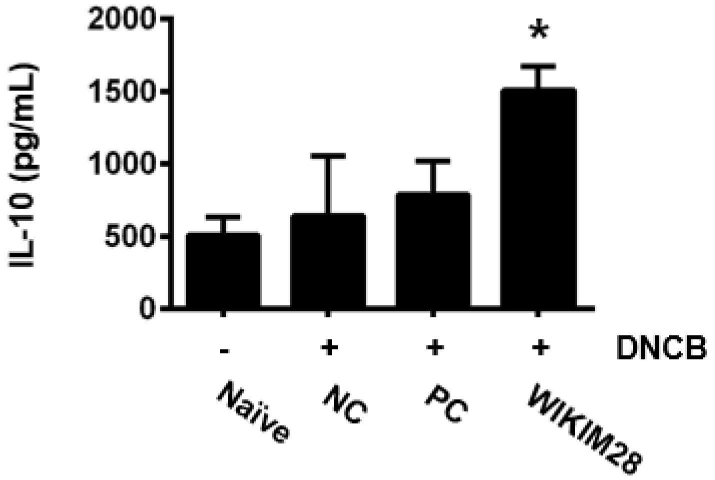

Supplementary Figure S1. ***W. cibaria*** **WIKIM28 increases the production of IL-10 in splenocytes of DNCB-induced AD mice.**

Splenocytes were re-stimulated with anti-CD3/CD28 mAbs for 1 day, and the levels of IL-10 in the supernatant were measured. Cytokine in the supernatant were detected by a cytometric Bead Array kit. Error bars indicate SE. Data are representative of three independent experiments. *$p < 0.05$, compared to the naïve group.

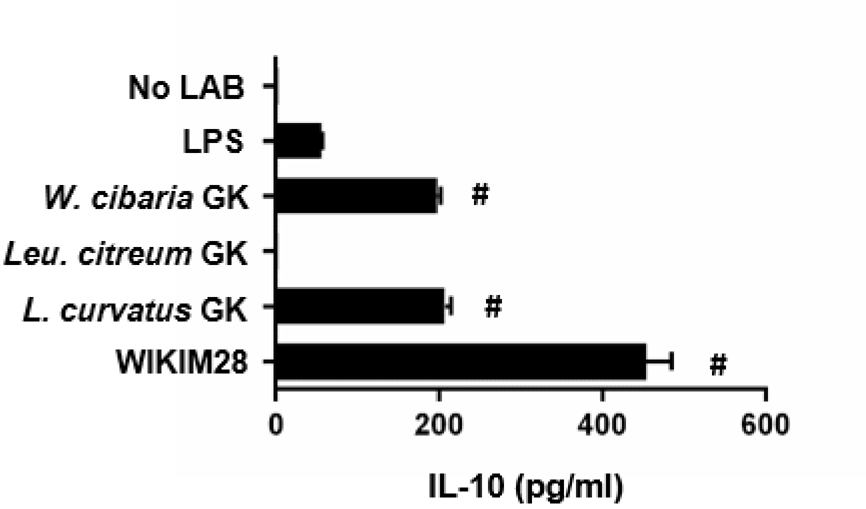

Supplementary Figure S2. *W. cibaria* WIKIM28 increases the production of IL-10 in BMDCs.

The release of IL-10 by BMDCs in response to LPS (100 ng/mL) or LAB for 24 h was measured. Cytokines in the supernatant were detected by a Cytometric Bead Array kit. Error bars indicate SE. Data are representative of three independent experiments. $^{\#}p < 0.001$, compared to the negative control group (No LAB). *W. cibaria* WIKIM28 was compared with LAB isolates isolated from gatkimchi (GK) (*W. cibaria* GK, *Weissella cibaria*; *Leu. citreum* GK, *Leuconostoc citreum*; *L. curvatus* GK, *Lactobacillus curvatus*).
